# Vitiligo

**DOI:** 10.1111/ddg.15706

**Published:** 2025-08-11

**Authors:** Markus Böhm, Adrian Tanew

**Affiliations:** ^1^ Department of Dermatology University Hospital Münster Münster Germany; ^2^ Private Office Vienna Austria

**Keywords:** comorbidity, Janus kinase inhibitors, phototherapy, shared decision makting, systemic therapy, topical therapy

## Abstract

Vitiligo is a common pigment disorder of the skin resulting in destruction of melanocytes. Non‐segmental vitiligo (NSV) is an autoimmune disorder. The etiopathogenesis of segmental vitiligo (SV) remains incompletely understood. Genetic predisposition and increased vulnerability of melanocytes towards stressors lead to a melanocyte‐specific CD8^+^ T cell‐driven immune response with a γ‐interferon signature. Vitiligo may lead to significant impairment of life quality. Importantly, vitiligo can be associated with somatic and psychological disorders. Early recognition, correct classification, precise assessment of disease extent and activity, burden of disease and presence of comorbidities is crucial for a holistic therapeutic management. Shared decision making with the patient should define treatment goals including halting disease progression, induction of repigmentation, prevention of relapses, and in rare cases depigmentatation of residual normal skin. Topical treatments in addition to corticosteroids and calcineurin inhibitors now include the Janus kinase inhibitor ruxolitinib cream, as a first‐line therapy officially approved for children from 12 years on and adults with NSV and facial involvement. Targeted phototherapies, in combination with topical corticosteroids or calcineurin inhibitors, are used for limited NSV or SV. For extensive NSV, whole‐body UVB‐NB phototherapy remains a cornerstone treatment and may be combined with oral corticosteroid mini‐pulses in rapidly progressive cases. Among emerging therapeutic options for NSV, oral Janus kinase inhibitors are the most advanced in clinical development.

## INTRODUCTION

Vitiligo is an acquired, usually chronic disease causing the loss of the pigment melanin in the skin. In recent years, significant progress has been made with respect to understanding of its pathophysiology resulting in new promising therapeutic options.^1^ Moreover, new epidemiological investigations and studies on disease burden and prevalence of associated diseases call for a broader view on the disease of vitiligo with the aim of a holistic and patient‐centered care.^2^

### Epidemiology

Depending on study and analyzed countries (USA, Europe, Japan), the prevalence ranges from 0.5% to 3.1%.^3^, ^4^ In a recently published study including both data of a large statutory health insurance company and primary data of a large cohort of subjects examined by dermatologists, a prevalence from 0.17% to 0.77% was estimated for Germany.^5^

### Etiopathogenesis

**FIGURE 1:**
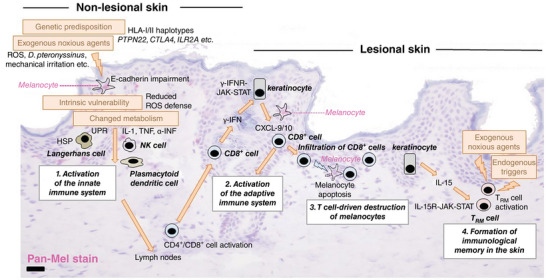
Pathophysiology of non‐segmental vitiligo.

Non‐segmental vitiligo (NSV), the main subtype of the disease, is generally considered an autoimmune disease (Figure 1).^6^, ^7^ The basis for the activation of the misdirected cellular immune response against melanocytes is a genetic predisposition that is well‐documented by a series of genome‐wide association studies.^8^ In these studies, more than 50 susceptibility loci increasing the risk of developing NSV were identified. The susceptibility genes include genes orchestrating the metabolism of pigment cells, such as *TYR* or *OCA2*, which are relevant for melanocyte‐specific antigen presentation, as well as a variety of genes regulating the innate and adaptive immune system, such as *NLPR1, IRF3, PTPN1, CTLA4, IL2RA, HLA‐DRB1, FOXP3*, or *FASL*.^9^ Some of these genes have also been identified as susceptibility genes in other autoimmune diseases, thus explaining the association of NSV with several autoimmune diseases (see below).

Non‐segmental vitiligo (NSV), the main subtype of the disease, is generally considered an autoimmune disease. Basis for the activation of the misdirected cellular immune response against melanocytes is a genetic predisposition that is well‐documented by a series of genome‐wide association studies.

The polygenetic predisposition and intrinsic abnormalities of melanocytes, for example in the redox system with impaired defense against oxidative stress, cause an increased vulnerability of the cells to exogenous noxious agents, such as mechanic irritation, pro‐oxidative irritants, or dust‐mite proteins.^10^, ^11^, ^12^ The resulting detachment of melanocytes from the epidermal tissue (melanocytorrhagy) has been reproduced in *ex vivo* experiments. This process is caused by impaired expression and distribution of the anchor protein E‐cadherin in melanocytes and is dependent on matrix metalloproteinase 9.^13^, ^14^, ^15^ Expression of *danger‐associated molecular patterns* (DAMPs) and presentation of melanocytic antigens results in activation of the innate immune system with activation of plasmacytoid dendritic cells, NK cells and innate lymphoid cells.^6^, ^15^, ^16^, ^17^

In the second phase, activation of the adaptive immune system results in formation of an “immunological synapse” between activated, infiltrating melanocyte‐specific CD8^+^ T cells with γ‐interferon (γ‐IFN) signature, epidermal keratinocytes and melanocytes. Secretion of γ‐IFN from CD8^+^ T cells causes activation of the canonical JAK1/2‐STAT signaling pathway in epidermal keratinocytes with subsequent secretion of CXCL9/10.^18^ These chemokines attract additional CD8^+^ T cells resulting in a *vicious cycle* with progressive destruction of melanocytes and increasing depigmentation of the skin.^7^ The CD8^+^ T cell‐driven immune response against melanocytes can be suppressed by JAK1/2 inhibitors in animal models of vitiligo and is the rationale for the current therapeutic strategies with topical and systemic JAK inhibitors. Interestingly, fibroblasts also participate in the immunopathogenesis of the disease and their regionally different γ‐IFN signatures can partially explain the symmetrical pattern of vitiligo lesions and the resistance to therapy in certain areas of the body.^19^

Secretion of γ‐IFN from CD8^+^ T cells causes activation of the canonical JAK1/2‐STAT signaling pathway in epidermal keratinocytes with subsequent secretion of CXCL9/10.

In the stable phase of NSV, CD8^+^ tissue‐resident memory cells (TRM) are thought to be responsible for the cutaneous disease memory and the tendency of NSV recurrence.^20^ Activation of these cells to cytotoxically active cells is dependent on interleukin (IL)‐15. In addition, this cytokine transmits intracellular signals via the JAK‐STAT signaling pathway.^21^

In the stable phase of NSV, CD8^+^ tissue‐resident memory cells (TRM) are thought to be responsible for the cutaneous disease memory and the tendency of NSV recurrence.

In contrast, the etiopathogenesis of segmental vitiligo (SV) is less well understood. It is assumed that a localized immunopathogenetic mechanism results in regionally and temporarily limited destruction of melanocytes.^22^ There are immunological differences concerning the levels of stress proteins and the number of circulating regulatory T cells between SV and NSV.^23^ Recently, histopathological, immunohistochemical, and ultrastructural evidence was found for the presence of varicella zoster virus in actively spreading SV, in addition to a somatic mosaic and neurogenic factors.^24^

### Clinical appearance

Clinically, vitiligo is characterized by white macules with potential depigmentation of hair within vitiligo lesions (leukotrichia, poliosis). Leukotrichia in vitiligo lesions is considered an unfavorable prognostic sign, given that the follicular stem cell reservoir of melanocytes is also destroyed in this case.^25^ Depending on the outdoor behavior, an increased sensitivity to sunlight in the skin areas affected by vitiligo due to reduction or absence of melanin pigment is reported. Up to 20% of affected individuals report lesional pruritus, usually indicating an active disease.^26^ In active disease, isomorphic irritant effects are found, for example, after mechanical irritation or in scar areas (Koebner phenomenon); especially in darker skin types, the borders of vitiligo lesions may be incompletely depigmented (hypochromic borders) and present with several shades of color (trichrome vitiligo). In rare cases, erythema may develop at the border of the white macules, sometimes even with palpable infiltration (inflammatory vitiligo). Additional signs of active vitiligo include minute disseminated lesions, so‐called confetti‐like lesions. While melanocytic nevi may present with perilesional depigmentation (halo nevi), this may occur also independently of vitiligo.

In the current classification, vitiligo is divided into three main types with subsets. The majority of all patients can be allocated to NSV.

**FIGURE 2:**
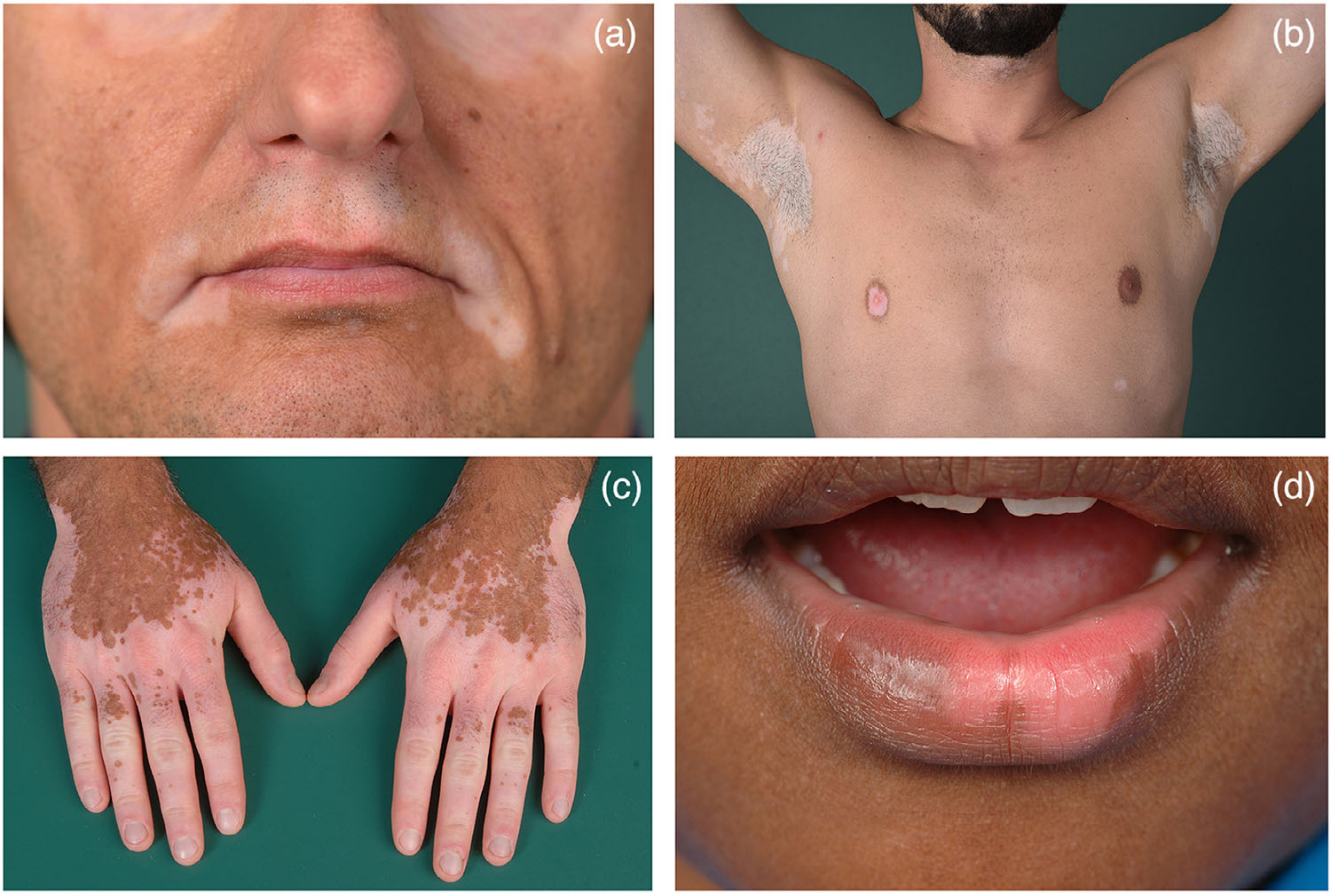
Clinical picture of non‐segmental vitiligo. Symmetrical distribution of white macules periorificially (a), on the trunk and in the axillae (b), on the extremities and dorsa of the hands (c) and on the vermilion area of the lower lip (d).

In the current classification, vitiligo is divided into three main types with subsets (Table 1).^27^ The majority of all patients can be allocated to NSV, with the common feature of symmetrically distributed white macules with predilection in the face, especially the periorificial area, and the anogenital region, on the extensor sites of extremities including wrists and ankles, dorsal aspects of hands and feet, as well as dorsal fingers and toes (Figure 2a–d).

**TABLE 1:** Clinical subtypes of vitiligo.

**Non‐segmental vitiligo (NSV)** Acrofacial Mucosal (more than one site) Generalized Universal Mixed (NSV, associated with SV) Rare variants: *Hypochromic vitiligo* *Follicular vitiligo* *Vitiligo punctata* **Segmental vitiligo (SV)** Unisegmental Bisegmental Polysegmental **Unclassifiable/undetermined vitiligo** Focal (one site) Mucosal (one site)

**FIGURE 3:**
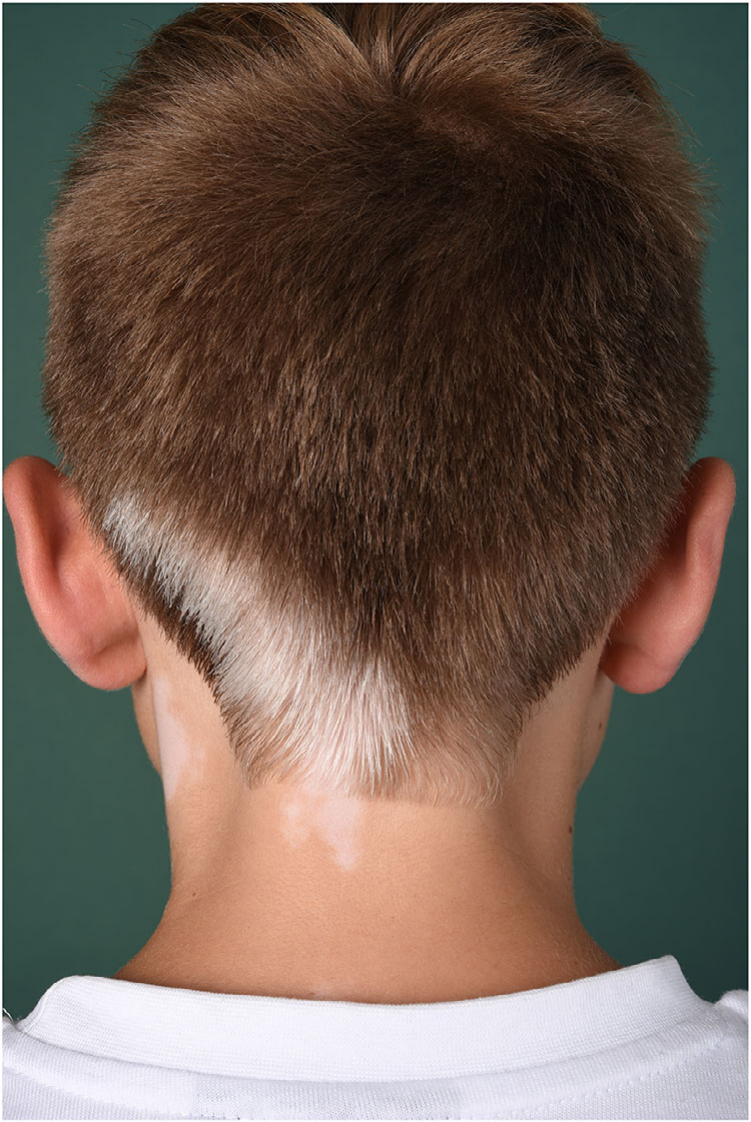
Clinical manifestation of segmental vitiligo. Unilaterally distributed white macules in the occipital region in the dermatomes C2‐C4 with leukotrichia.

The much less common SV accounts for 5%–16% of all vitiligo cases^28^, ^29^ and is characterized by unilaterally localized white macules with early leukotrichia. Unisegmental, bisegmental, and polysegmental involvement has been observed (Figure 3). Concomitant presence of unilateral and symmetrically distributed white macules is referred to as mixed vitiligo, which is allocated to NSV due to its prognosis.

These forms should be differentiated from the unclassifiable (undetermined) form with individual lesions on the body or mucous membrane that may unmask itself as SV or NSV based on the development of additional lesions over the course of 1–2 years (Table 1). Less common special forms of NSV include follicular vitiligo, hypochromic vitiligo in patients with dark skin (not to be confused with hypochromic borders as sign of active vitiligo), and vitiligo punctata (Table 1).^27^

The clinical differentiation in NSV and SV is important, given that NSV has an unpredictable course with frequent recurrence and is associated with other inflammatory diseases (autoimmune vitiligo).^30^ While NSV has a bimodal age distribution with peaks at the age of 10.3 and 34 years,^31^ SV often occurs in childhood. Leukotrichia develops early in its course. SV is typically not associated with other autoimmune diseases, and often stops spontaneously after 1–2 years.^22^, ^30^

### Differential diagnosis

Depending on the type and extent of vitiligo, this includes inherited and acquired localized, generalized, and universal depigmentation disorders (reduction or absence of melanocytes). Differential diagnoses are listed in Table 2. In cases of doubt, histological clarification is useful, for example for differentiation of progressive macular hypomelanosis (in Wood light, often follicular orange fluorescence) and other rare leukodermas. A previous pityriasis versicolor with persisting hyomelanotic macules (pityriasis versicolor alba) may be confused with vitiligo. In some cases, melasma may be misinterpreted as extensive facial vitiligo with residual pigmentation.

**TABLE 2:** Differential diagnosis of vitiligo.

**Congenital/genetic/syndromal** Nevus anemicus Nevus depigmentosus Piebaldism Tuberous sclerosis Klein‐Waardenburg syndrome Hypomelanosis of Ito Albinism Hermansky‐Pudlak syndrome Menkes syndrome Griscelli syndrome Ziprkowski‐Margolis syndrome **Postinflammatory/postinfectious** Pityriasis alba Pityriasis versicolor Sarcoidosis Psoriasis Leishmaniasis Syphilis Leprosy Onchocerciasis **Associated with neoplasms** Melanoma Mycosis fungoides **Idiopathic** Progressive macular hypomelanosis Idiopathic guttate hypomelanosis **Drug‐related** Interferon Imiquimod Checkpoint inhibitors Alemtuzumab Imatinib Cyclin‐depedent kinase 4 and 6 inhibitors **Induced by chemicals** Phenols und phenol derivatives **Other causes** Extragenital lichen sclerosus Eruptive hypomelanosis

### Associated disorders and diseases

Vitiligo can be associated with a variety of diseases. In this context, somatic and psychological comorbidities should be distinguished.

### Somatic comorbidity

Against the background of genetic predisposition of NSV to autoimmune diseases, an increased prevalence for other autoimmune diseases should be expected in this vitiligo subtype, in particular. In the available studies, however, NSV and SV were not always clearly differentiated. According to a recently published review, an increased prevalence of thyroid diseases, alopecia areata, and psoriasis vulgaris was observed most often in vitiligo patients compared to the general population.^32^ A US‐American study of 1,098 patients with vitiligo and a 10‐year follow‐up showed that 20% of all patients with vitiligo have at least one other autoimmune disease.^33^ The most commonly associated autoimmune diseases were thyroid diseases (12.3%), followed by alopecia areata (3.8%). These results support earlier findings from studies with patients of European ethnicity with generalized vitiligo from the USA and England (thyroid diseases in 17% of the examined individuals), whereas a study from Taiwan with 14,883 patients demonstrated only a marginally increased prevalence of thyroid diseases.^34^ A meta‐analysis of 37 studies and 78,714 patients with vitiligo found prevalences with a pooled *odds ratio* (OR) of 3.93 (95% confidence interval [CI]: 2.23–6.93) for thyroid diseases, 5.88 (95% CI: 2.68–12.89) for autoimmune thyroid diseases, 3.38 (95% CI: 2.97–4.96) for anti‐thyroid peroxidase (TPO) antibodies and 3.51 (95% CI: 2.35–5.26) for anti‐thyroglobulin (TG) antibodies.^35^ The prevalence of thyroid diseases and anti‐TPO antibodies was significantly higher in patients with NSV compared to patients with SV and was also correlated with the extent of NSV.

Against the background of genetic predisposition of NSV to autoimmune diseases, an increased prevalence for other autoimmune diseases should be expected in this vitiligo subtype, in particular.

According to a recently published review, an increased prevalence of thyroid diseases, alopecia areata, and psoriasis vulgaris was observed most often in vitiligo patients compared to the general population.

Epidemiological studies have also confirmed the association between vitiligo and diabetes mellitus or components of the metabolic syndrome. A systematic review with meta‐analysis of nine case‐control‐studies (overall, 15,657 vitiligo patients) revealed both an association of vitiligo with diabetes mellitus type I (pooled OR: 2.90; 95% CI: 1.53–5.48; p = 0.001) and diabetes mellitus type II (pooled OR: 2.37; 95% CI: 1.71–3.28; p < 0.001).^36^ These results are corroborated by another meta‐analysis on comorbidity of components of the metabolic syndrome. In the 30 analyzed studies (overall, 28,325 vitiligo patients), significant associations with diabetes mellitus (pooled OR: 3.30; 95% CI: 2.10–5.17) and obesity (pooled OR: 2.08; 95% CI: 1.40–3.11) were found. The pooled prevalence of arterial hypertension in the patients was 19.0% (95% CI: 2.0%–36.0%).^37^

In some cases, NSV may be a component of autoimmune polyglandular syndromes (APS). Systematic analyses of large cohorts of vitiligo patients on the prevalence of APS are, however, lacking. APS3 and 4 are probably the most common.^38^

Interestingly, two recent epidemiological studies from Brazil and Taiwan showed increased rates of sensorineural hearing loss in patients with vitiligo.^39^, ^40^ In the elaborate Taiwanese study with 12,048 patients and 52,192 controls, a 2.2‐fold increased risk of developing this hearing disorder was found. These data must be confirmed in other populations.

### Psychological comorbidity

Similar to other dermatoses, vitiligo can cause significant stigmatization of affected patients with considerable impairment of the quality of life (QoL).^41^ This, in turn, forms the basis for the development of psychological disorders and diseases. Many studies show that the QoL, measured by *Dermatology Life Quality Index* (DLQI) – the globally most commonly used *Patient‐Reported Outcome Measure* (PROM)^42^, is impaired in patients with vitiligo. The involvement of visible skin areas (face and hands), but also of the genital region, as well as young adult age and degree of disease extent in percent of body surface area (BSA), are associated with a higher DLQI (higher life quality impairment). Skin color, relationship status, and geographic region also play a role.^42^, ^43^

Similar to other chronic skin diseases, vitiligo can cause significant stigmatization of affected patients with considerable impairment of the quality of life.

Recent systematic reviews and meta‐analyses also show that vitiligo is associated with psychosocial and psychological comorbidity.^44^, ^45^, ^46^, ^47^ Most of the 168 studies included in the most recent review reported depressive symptoms and depressive disorders (41 studies) and anxiety disorders (20 studies).^47^ Other identified psychological disorders included adaptation disorders (12 studies), suicidal ideation (8 studies), sleep disturbance (7 studies), obsessive‐compulsive disorders (5 studies), and somatoform disorders (3 studies). In addition, psychosocial burden and accompanying symptoms are often observed. Ten studies reported on relationship problems including sexual dysfunction, eight studies on stigmatization. Moreover, avoidance behavior (9 studies), reduced self‐consciousness (8 studies), anger (6 studies), alexithymia (4 studies), and low self‐esteem (4 studies) have been identified in vitiligo patients. Depending on study design and analyzed geographic region, however, large ranges in the prevalences of these psychosocial and psychological comorbidities were found.^47^

### Diagnostic workup

**FIGURE 4:**
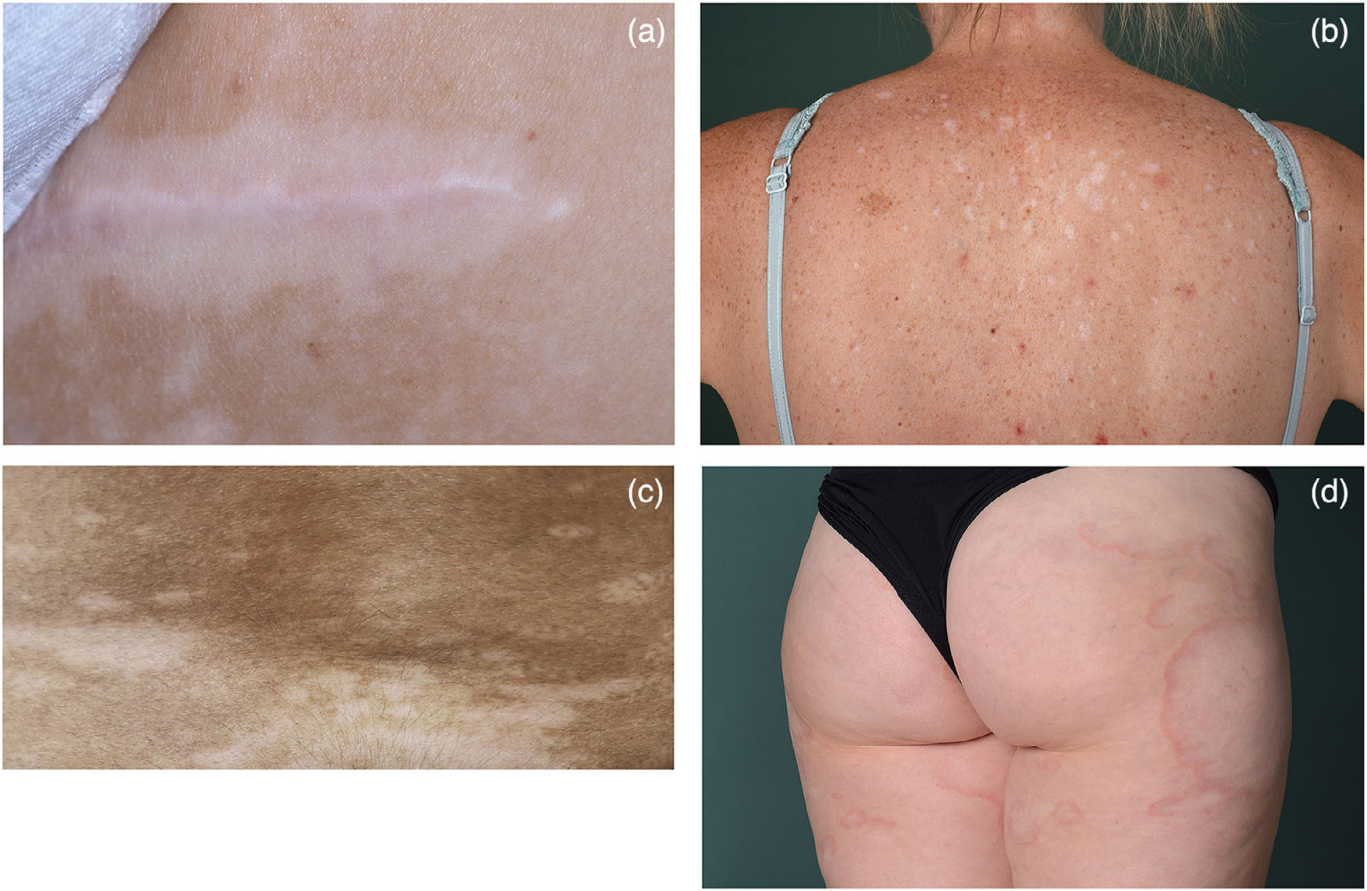
Clinical signs of active vitiligo. (a) Koebner phenomenon localized in a scar, (b) confetti‐like vitiligo lesions; may be confused with extragenital lichen sclerosus but lack thickening of the skin, (c) hypochromic borders and (d) inflammatory vitiligo.

The diagnosis of vitiligo is usually made clinically. Examination with Wood light facilitates the identification of vitiligo lesions, especially in affected individuals with fair skin type or in those with confetti‐like lesions.^48^ All patients should be examined for clinical signs of active disease (Koebner phenomenon, hypochromic borders, inflammatory vitiligo, and confetti‐like lesions) (Figure 4). Koebner phenomenon has been identified as most valid criterion of active disease.^49^ Photographic documentation may improve the assessment of activity and extent, if it is performed in a standardized manner throughout the disease.^50^

The diagnosis of vitiligo is usually made clinically. Examination with Wood light facilitates the identification of vitiligo lesions, especially in affected individuals with fair skin type and those with confetti‐like lesions.

Validated scores are available to determine the extent of vitiligo allowing conclusions with respect to the disease activity over time.

Validated scores are available to determine the extent of vitiligo allowing conclusions with respect to the disease activity (active, non‐active, stable) over time (Table 3).^51^, ^52^, ^53^, ^54^, ^55^ Central element of all measurement tools is the assessment and documentation of the affected body surface area in percent. This is also a key aspect for treatment planning. The entire palm of the patient including the volar sides of the fingers corresponds to 1% BSA, the index finger to 0.1% BSA, the distal segment of the index finger to 0.03% BSA.^56^ There is no generally accepted classification of the affected body surface area in terms of degrees of severity; a classification obtained from the perspective of patients is as follows: mild ≤ 1.05% BSA, moderate > 1.05–6.45% BSA, and severe > 6.45% BSA.^57^ The most commonly used score in clinical trials is the *Vitiligo Area Scoring Index* (VASI), which comprises not only the assessment of the affected body surface area, but also the degree of depigmentation within the affected skin areas.^51^ In clinical trials, the VASI is often reported separately only for the face (F‐VASI) and for the total body (T‐VASI). While the score described by the *Vitiligo European Task Force* (VETF) determines also the affected body surface area and degree of depigmentation, it records also the disease activity.^52^ The *Vitiligo Extent Score* (VES) is based on the assessment of the affected body surface area by means of comparison pictures^53^ and can be used via an app (https://www.vitiligo‐calculator.com/) by both the examiner and the patient (*Self‐Assessment Vitiligo Extent Score*, SA‐VES).^54^ VES and SA‐VES show an excellent correlation between each other.^54^

Given that NSV, in particular, is associated with autoimmune diseases, the presence of latent autoimmune thyroiditis should be excluded in patients with vitiligo and negative history.

**TABLE 3:** Scoring methods for evaluation of vitiligo extent.

Score	Acronym	Reference
Vitiligo Area Scoring Index	VASI	Hamzavi, et al. 2004
Vitiligo European Task Force assessment	VETFa	Taieb, et al. 2007
Vitiligo Extent Score	VES	van Geel, et al. 2016
Self‐Assessment Vitiligo Extent Score	SA‐VES	van Geel, et al. 2017
Vitiligo Extent Score plus	VESplus	van Geel, et al. 2018

Given that NSV, in particular, is associated with autoimmune diseases, the presence of latent autoimmune thyroiditis should be excluded in patients with vitiligo and negative history by determination of thyroid‐stimulating hormone (TSH), antibodies against TPO and TG, as well as antibodies against thyrotropin receptor.^58^, ^59^, ^60^ In case of a respective history or clinical evidence, a further diagnostic workup to exclude additional diseases, for example, atopic diseases, diabetes mellitus, or pernicious anemia, is appropriate. Broad routine screening for autoantibodies is not recommended.

In clinical studies, the DLQI is usually used as PROM for screening of quality of life and disease burden of affected patients with vitiligo, although it is not specific for this disease. In contrast, Vitiligo (Viti)QoL^61^ and *Vitiligo Impact Patient Scale* (VIP)^62^ are more suitable as validated PROMs. Use of these PROMs provides important information on further treatment planning and the possible choice of supportive therapies (see below).^58^, ^59^

### Therapeutic options

**FIGURE 5:**
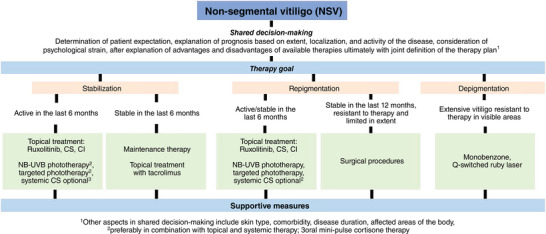
Simplified scheme for the treatment of the most common form of vitiligo, nonsegmental vitiligo (NSV) (modified from ^63^). *Abbr*.: CS, corticosteroids; CI, calcineurin inhibitors. Modified from ref. ^63^.

The therapy is based on subtype of vitiligo, age and skin phototype according to Fitzpatrick, extent and activity of the disease, present comorbidities, and individual level of psychological strain of those affected.^58^, ^59^, ^63^ The therapeutic goals should be defined together with the patient: *(1)* stop of disease progression, *(2)* induction of repigmentation *(3)* maintenance of achieved repigmentation (stabilization). Only in exceptional cases may *(4)* depigmentation be considered (Figure 5). Given that this treatment results in permanent destruction of melanocytes in unaffected healthy skin, it is our view that this goal should be understood as the last resort in individual cases, only after failure of all available therapies, especially in European skin types.

The therapeutic goals should be defined together with the patient: *(1)* stop of disease progression, *(2)* induction of repigmentation *(3)* maintenance of achieved repigmentation (stabilization). In exceptional cases only, *(4)* depigmentation may be considered.

## TOPICAL THERAPIES

### Topical corticosteroids

Due to their broad but non‐specific anti‐inflammatory effect, topical corticosteroids (TCS) have been used for decades for the treatment of vitiligo in order to stabilize the disease and/or achieve repigmentation. Topical corticosteroids are considered a potential first‐line therapy in case of limited extent and extrafacial involvement, in particular.^58^, ^63^ The rationale for monotherapy with TCS in vitiligo is based on older and usually smaller studies (< 100 patients). However, the design of these studies does not meet the current standard of clinical studies and their results are only comparable to a limited degree with those of the most recent randomized controlled trials (RCTs). In a meta‐analysis of patients with localized vitiligo (< 20% BSA), a pooled extent of 55% with respect to achieved 75% repigmentation was calculated for class IV TCS (clobetasol 2‐propionate). There was no significant difference to compounds of class III, such as betamethasone 17‐valerate (56%).^64^ However, this meta‐analysis also included results from non‐randomized controlled trials, studies with TCS of different potency, and different duration of application (2–21 months). The response rates obtained in more recent RCTs comparing TCS and topical calcineurin inhibitors seem to be more realistic (see below). Similar to all vitiligo therapies, repigmentation by TCS is predominantly expected in the face and neck region, while trunk, extremities, and acral areas, as well as those with leukotrichia usually show a poorer response. Due to the known adverse effects of moderate and potent TCS (atrophy, telangiectasia, hypertrichosis, acneiform lesions) in case of longer application, the use of older TCS is limited to several months. Particularly in the face (especially the eyelid area) and in the intertriginous areas of children (risk of resorption), they should be used with caution. To avoid adverse effects, modern class III TCS with low atrophogenic risk, such as mometasone furoate, should be used. There are, however, no evidence‐based data concerning the optimal application regimen of TCS in vitiligo. A proposed therapeutic regimen consists of application for 3 months once daily or alternatively for a maximum of 6 months once daily with 2 weeks of treatment followed by an interval of 2 weeks.^58^, ^63^ In case of repigmentation in areas without side effects, however, this therapy may be prolonged.

Topical corticosteroids are considered a potential first‐line therapy in case of limited extent and extrafacial involvement, in particular.

### Topical calcineurin inhibitors

The topical calcineurin inhibitors (TCI) tacrolimus and pimecrolimus are also considered first‐line therapies for adult patients and children with limited vitiligo, especially in the face and neck region, as well as intertriginous areas, where the application of topical corticosteroids is problematic. The repigmentation effect of TCI is attributed to their non‐specific anti‐inflammatory effect and to a migration‐promoting effect on melanocytes. However, both substances are only approved for atopic dermatitis. Their application in vitiligo represents an *off‐label* use and is, therefore, generally not reimbursable. While the repigmentation effect of TCS and TCI in the face is similar (in this area, however, TCS are not recommended due to their adverse effects), corticosteroids appear to be superior to TCI on other areas of the body.^65^, ^66^, ^67^ In a meta‐analysis, repigmentation of at least 25% was observed in 55% of patients, and repigmentation of at least 75% in 18.1% of patients. However, the patients had received monotherapy with TCI only for a median period of 3 months.^68^ 35.4% of the treated children exhibited repigmentation of at least 75% in the face and neck region. Occlusion may enhance the effect of monotherapy with TCI.^69^ While application twice daily for at least 6–9 months is recommended,^70^ TCI may be applied for a longer period in case of good response without side effects. TCI are also suitable for maintaining pigmentation after successful treatment. In a small RCT, application of 0.1% tacrolimus ointment twice weekly for 24 weeks could reduce the recurrence risk from 48.4% (placebo) to 26.8%.^71^ The most common adverse effects of TCI include pruritus, burning sensations, and erythema in the application area (among others factors, also in case of alcohol consumption).

The topical calcineurin inhibitors tacrolimus and pimecrolimus are also considered first‐line therapies for adult patients and children with limited vitiligo, especially in the face and neck region, as well as intertriginous areas.

### Topical ruxolitinib

With the European approval of the JAK‐1/2 inhibitor ruxolitinib in April 2023, a first‐line therapy in form of a 1.5% cream is available for the first time for patients aged 12 years or older with NSV and facial involvement. Ruxolitinib cream can be applied twice daily on up to 10% of the body surface area without reaching systemically relevant effective levels.^72^ Given that pregnancy and lactation are contraindications for the use of ruxolitinib cream, contraception throughout its application is recommended for female patients of childbearing age. If repigmentation of at least 25% of the treated lesions is observed after one year, the therapy may be continued. Application of the cream on mucous membranes is prohibited. The approval is based on a placebo‐controlled phase II dose‐finding trial demonstrating that the cream has the highest effect when applied twice daily at a concentration of 1.5%,^73^ and on two subsequent placebo‐controlled phase III trials (TRueE‐V1 and TRueE‐V2) on a total of 674 patients.^74^ After treatment for 52 weeks, 50.3% of the patients achieved F‐VASI75, which is considered a clinically relevant improvement. Approximately 30% even achieved F‐VASI90. After 52 weeks of treatment, 51.1% of the patients achieved T‐VASI50. Both phase III trials showed high patient satisfaction, measured by *Vitiligo Noticeability Scale* and *Color‐Matching Response*. The best response to ruxolitinib cream was achieved in the head and neck region, followed by arms and legs, trunk, and, finally, hands and feet.^72^ No significant difference was observed between adults and children. Disease duration, potential comorbidities, or previous therapies had also no effect on the treatment response.^72^, ^74^ The most common adverse effects were acneiform lesions (5.9% and 5.7%) and pruritus at the application sites (5.0% and 5.3%); in no case, however, did they cause a discontinuation of therapy.^74^ In elongation studies, where ruxolitinib cream was applied for an additional 52 weeks, a further increase in the proportion of patients with clinically relevant F‐VASI75 to 66% was observed.^72^ Compared to TCS and TCI, the therapy with topical ruxolitinib is relatively expensive. However, the overall economic efficiency must always be assessed against the background of available, approved and equivalent alternatives and long‐term consequences like the potential development of psychological comorbidities in case of insufficient therapeutic efficacy. As described above, TCS are inappropriate for long‐term therapy especially in the facial area while the therapy with topical TCI is *off‐label*.

With the European approval of the JAK‐1/2 inhibitor ruxolitinib in April 2023, a vitiligo‐specific first‐line therapy in form of a 1.5% cream is available for the first time for patients aged 12 years or older with NSV and facial involvement.

### Phototherapies

Currently, predominantly narrow‐band ultraviolet B (NB‐UVB), outside of Europe also photochemotherapy (PUVA), are used.^75^ The mode of action of phototherapies is based on the pleiotropic immunomodulatory effects of UV radiation and the pronounced stimulation of the pigment system.^76^, ^77^, ^78^, ^79^ Apart from the depletion of epidermal Langerhans cells, NB‐UVB counteracts the activation of Th17 cells and results in a reduction of IL‐17 and IL‐22 as well as a greatly reduced JAK1 expression. Moreover, there is an upregulation of IL‐10 resulting in the induction of regulatory T cells. Analyses of circulating CD3^+^ CD8^+^ CD28^+^ T cells and circulating CD4^+^ and CD8^+^ central memory T cells (T_CM_) showed significantly reduced levels on phototherapy.^80^, ^81^ At the same time, radiation with NB‐UVB stimulates the proliferation of melanocytes and the differentiation and migration of melanocytic stem cells in the outer root sheath of the hair and enhances melanogenesis.

The mode of action of phototherapies is based on the pleiotropic immunomodulatory effects of UV radiation and the pronounced stimulation of the pigment system.

### NB‐UVB monotherapy

Whole‐body NB‐UVB is primarily used to achieve repigmentation in patients for whom local therapy is no longer indicated due to the extent of vitiligo. Moreover, regular irradiation (2–3 times per week for 6 months) in patients with active and rapidly progressive vitiligo has the potential to halt disease activity.^82^, ^83^ The response to NB‐UVB phototherapy is highly variable and dependent on numerous factors, such as UV dosage and therapy duration, adherence to therapy, type of vitiligo, skin phototype, disease duration, localization of affected skin areas, absence of leukotrichia, and psychological factors. A meta‐analysis showed that ≥ 50% repigmentation and ≥ 75% repigmentation of the affected skin areas can be achieved in 56.8% and 35.7% of patients, respectively, after 12 months of radiation. These results can be further improved by combination with other therapies (see below). Phototherapy in vitiligo is usually performed two to three times per week and should be evaluated every 3 months.^84^ The minimum duration of treatment is 6 months, given that it may take more than 3 months until the onset of significant repigmentation.^85^, ^86^ The recurrence rates after 12 months of NB‐UVB therapy range from 21% to 45%, although these data are based on very low case numbers.^88^, ^89^

Whole‐body NB‐UVB is primarily used to achieve repigmentation in patients for whom local therapy is no longer indicated due to the extent of vitiligo.

### Combination therapies

In many studies, mostly small and uncontrolled, an attempt was made to improve the repigmentation achievable by NB‐UVB phototherapy by combination with topical or systemic therapies. With respect to additional application of topical corticosteroids, a randomized three‐arm trial (mometasone furoate ointment vs. NB‐UVB home therapy with a handheld device vs. combination) on 517 patients (of these, 370 were evaluable) showed only a minor superiority of the combination therapy after a study period of 9 months.^90^ The synergistic effects of pseudocatalase cream and NB‐UVB described by one work group^93^, ^94^ were not confirmed in later studies.^91^, ^92^ In contrast, a combination of NB‐UVB with both vitamin D derivatives^95^ and tacrolimus^68^, ^96^, ^97^ seems to be more effective than NB‐UVB monotherapy.

In mostly small and uncontrolled studies, an attempt was made to improve the repigmentation achievable by NB‐UVB phototherapy by combination with topical or systemic therapies.

Phototherapy with additional administration of oral corticosteroid mini‐pulses (see below) can achieve faster cessation of disease activity and a higher rate of repigmentation.^98^, ^99^, ^100^ While in a recent placebo‐controlled comparison study of NB‐UVB versus NB‐UVB plus dexamethasone mini‐pulses the patients in the combination group also showed a faster response, no differences between the two groups in terms of degree of repigmentation were found after 6 months.^101^

Based on the importance of oxidative stress in vitiligo development, several studies on the concomitant administration of systemic antioxidants with NB‐UVB phototherapy have been performed.^102^ Randomized controlled trials demonstrated an additive therapeutic effect for a mixture of lipoic acid, vitamin E, vitamin C, unsaturated fatty acids, and cysteine^103^, Polypodium leucotomos extract,^104^ vitamin E,^105^ and gliadin biopolymer‐protected superoxide dismutase.^106^ In a double‐blind, randomized trial, monotherapy with *Ginkgo biloba* was effective in stabilizing disease and promoting repigmentation.^107^ No data are available on the efficacy in combination with phototherapy.

### Adverse effects and risks

When performed correctly, NB‐UVB therapy is safe and has few side effects. The most common acute side effect is a mild to moderate UV erythema that occurs, similar to sunburn, within 24 hours of irradiation and requires only adjustment of the dosage. The photocarcinogenic risk of long‐term NB‐UVB treatment (> 100 exposures) must be considered in relation to the disease. Several studies are available showing, with exception of a Korean study,^108^ that vitiligo patients are at a lower risk of developing non‐melanoma and melanoma skin cancer than control subjects.^109^, ^110^, ^111^ Concerning the additional photocarcinogenic risk of NB‐UVB therapy in vitiligo patients, most studies show that NB‐UVB phototherapy does not result in a higher incidence of melanoma or non‐melanoma skin cancer.^112^, ^113^, ^114^ Only in one report from Singapore was a slightly increased incidence of non‐melanoma skin cancer found in vitiligo patients treated with NB‐UVB.^115^

Concerning the photocarcinogenic risk of NB‐UVB therapy in vitiligo patients, most studies show that NB‐UVB phototherapy does not result in a higher incidence of melanoma or non‐melanoma skin cancer.

### Targeted phototherapies

For targeted phototherapy, irradiation devices are employed that emit high‐intensity UVB light and are used in segmental vitiligo or stable limited non‐segmental vitiligo.^116^ This type of phototherapy is also used as adjuvant treatment of surgical procedures to optimize treatment outcomes^117^, ^118^ and can be used in combination with NB‐UVB for recalcitrant lesions.^119^ The most commonly used devices are xenon chloride excimer laser and xenon chloride excimer lamp, which is not based on laser technology. Both devices emit light at a wavelength of 308 nm.^120^, ^121^, ^122^

For targeted phototherapy, irradiation devices are employed that emit high‐intensity UVB light and are used in segmental vitiligo or stable limited non‐segmental vitiligo.

Compared to NB‐UVB treatment, repigmentation on excimer therapy occurs faster and uses lower cumulative UV doses.^120^, ^123^, ^124^ While a systematic review showed comparable efficacy of excimer laser and NB‐UVB with respect to achievement of ≥ 75% or 100% repigmentation, a higher rate of ≥ 50% repigmentation was achieved with the excimer laser.^125^ Based on the prevailing evidence, excimer laser and excimer lamp are equally effective in treatment of vitiligo.^122^, ^126^, ^127^

The recurrence rate within 1–3 years after excimer laser therapy is 2.6%–15%.^128^, ^129^ Follow‐up examination of children treated with excimer laser after 3.4 years on average showed localization‐dependent recurrence rates of 20% in the face, 60% on the body, and 80% on the hands.^130^ The combination with TCS or TCI increased the efficacy of targeted phototherapy and may enable a response in previously recalcitrant areas.^97^, ^131^, ^132^, ^133^, ^134^, ^135^, ^136^, ^137^, ^138^ Two reviews on the combination of excimer light and vitamin D analogs demonstrate no additive effect of topical vitamin D application^95^, ^135^ In a recent network meta‐analysis of eleven randomized trials, however, the combination with tacalcitol, calcipotriol or a *cosmeceutical* containing superoxide dismutase, copper, zinc, vitamin B12, and calcium pantothenate showed the highest probability for 75% repigmentation compared to monotherapy with excimer laser.^139^ Due to the low case numbers and the short study duration (≤ 4 months), however, these results should be interpreted with extreme caution.

### Immunosuppressive systemic therapies

To arrest the activity of vitiligo in rapidly progressive NSV or early SV, oral mini‐pulse therapy with corticosteroids should be considered in all patients.^58^, ^63^ The purpose of the therapy is the stabilization of active disease. Given that repigmentation is usually not induced, concomitant initiation of NB‐UVB treatment is recommended (see above). Administration of betamethasone (5 mg) or dexamethasone (2.5–5 mg) on two consecutive days in the week, followed by five days without therapy, for 3 to 6 months is recommended. Other corticosteroids can be administered in dose equivalents (methyl prednisolone at 5 times higher dose, prednisone and prednisolone at 6.25 times higher dose). In the available non‐controlled studies, a stabilization rate of > 80% by oral mini‐pulse (OMP) therapy has been reported for extensive, rapidly progressive NSV.^98^, ^140^, ^141^ While potential adverse effects, such as weight gain, mood swings, acneiform lesions, sleep disturbance, disturbed menstruation, hypertrichosis, growth retardation in children, and immunosuppression must be discussed with the patient in advance, they are usually not pronounced and rarely cause therapy discontinuation.

To arrest the activity of vitiligo in rapidly progressive NSV or early SV, oral mini‐pulse therapy with corticosteroids should be considered in all patients.

Other immunosuppressive and immunomodulatory substances, such as methotrexate, ciclosporin, azathioprine, or minocycline, showed heterogeneous effects on disease activity in smaller, non‐controlled studies. However, none of these therapies is generally recommended, and all represent an *off‐label* use in vitiligo.^58^, ^63^ Similarly, there is currently no evidence for the use of biologics. According to a large Korean, controlled long‐term study on 11,442 patients, therapy with tumor necrosis factor (TNF) inhibitors even increases the risk of developing vitiligo (*hazard ratio*: 1.99).^142^ These results are supported by a WHO pharmacovigilance database study linking not only TNF inhibitors but also *checkpoint* inhibitors, imiquimod, alemtuzumab, and ustekinumab with the induction of vitiligo.^143^

Other immunosuppressive and immunomodulatory substances, such as methotrexate, ciclosporin, azathioprine, or minocycline, showed heterogeneous effects on disease activity in smaller, non‐controlled studies.

### Surgical interventions

Surgical procedures are primarily used in SV and focal NSV not responding to conservative therapy.^144^, ^145^ Usually, the absence of disease activity in the preceding 12 months and the absence of the Koebner phenomenon are used as criteria for stability of vitiligo (stable vitiligo).^59^, ^63^, ^146^ Autologous grafting of tissue (full‐thickness skin in form of small skin punches, split‐thickness skin, or epidermis obtained by suction blistering) and non‐cultured or cultured melanocytic or epidermal cell suspensions are distinguished.^145^, ^147^, ^148^, ^149^ Due to the cell expansion, cultured cell suspensions have the advantage that they allow for the treatment of significantly larger areas with a donor‐recipient ratio of up to 1 : 60.^150^ However, their preparation is technically demanding, time‐consuming, and expensive. Since 2005, a commercial kit for the preparation of autologous cell suspensions (RECELL^®^) is available as a medical device on the European market that can be used for surgical interventions in vitiligo in addition to the treatment of burn injuries. In America, this kit was approved by the FDA for the treatment of patients aged 18 years or older with stable vitiligo only in June 2023.

Surgical procedures are primarily used in segmental vitiligo and focal non‐segmental vitiligo not responding to conservative therapy.

The de‐epithelialization of the recipient area prior to grafting can be performed by dermabrasion, fractionated or ablative laser treatment, or suction blister technique. One decisive factor for the choice of the grafting method is the localization of vitiligo areas. Surgery in certain anatomical regions (lips, eyelids, genital area, fingers, and toes) is challenging.^144^, ^151^, ^152^, ^153^ Depending on the applied technique, potential side effects include postoperative secondary infections, color mismatch between repigmented vitiligo area and surrounding skin^145^ ^.^ ^154^, Koebnerization, scar formation in the donor area, cobblestone‐like surface structures in the treated area after grafting of skin punches, and milia.^145^, ^154^, ^155^

The efficacy of the different grafting methods was evaluated in a recent meta‐analysis including 117 studies.^156^ More than 90% repigmentation after treatment was achieved, on average, in 72% of patients after split‐thickness skin grafting, 61.7% after grafting of epidermis obtained by suction blister technique, 56.8% after grafting of cultured epidermal cells, 47.5% after grafting of non‐cultured epidermal cells, and 45.8% after grafting of skin punches. It should be noted that the results of the various studies were very heterogenous. Apart from the expertise of the dermatosurgeon, younger patient age, localization of recipient areas, and type of vitiligo are decisive factors for the therapeutic success (better response of SV compared to NSV).^157^ Although older studies suggest that adjuvant NB‐UVB phototherapy of the recipient area will further improve the therapeutic outcome,^158^, ^159^, ^160^ this effect was not confirmed in the meta‐analysis mentioned above.

Apart from the expertise of the dermatosurgeon, younger patient age, localization of recipient areas, and type of vitiligo are decisive factors for the therapeutic success.

There are only limited data available on the persistence of the achieved repigmentation after surgical intervention. Increased recurrence rates were found in case of insufficient disease stability, poor repigmentation after grafting, and NSV.^154^, ^158^, ^161^ Follow‐up for 6 and 5 years after grafting of non‐cultured epidermal cell suspensions, respectively, showed unchanged and ≥ 75% repigmentation in more than half of the patients.^162^, ^163^ Given that it may take up to 2 years after surgical intervention until maximum repigmentation is achieved, another grafting attempt in case of inadequate response should only be performed after waiting for an appropriate period.^163^

### Supportive measures

#### Shared decision‐making

Shared decision making offers genuine participation of the patient with the caregiver during the whole therapeutic management. Visual tools (shared decision‐making tools) can provide here disease‐specific background information and information on advantages and disadavantages of any treatment option (digital or in print for patients. This can be very useful for therapeutic decision‐making based on jointly defined therapy goals (see above). So far, however, *shared decision‐making tools* for vitiligo have only recently been investigated.^164^

### Sunscreen

Given the absence of photoprotective eumelanin, the depigmented skin of vitiligo patients is more sensitive to UV radiation. Accordingly, the use of highly potent external sunscreens (sun protection factor [SPF] ≥ 50) is recommended.

### Psychological and psychosocial support

The DLQI is routinely used in dermatology and allows for a first screening for psychosocial impairment in vitiligo patients. However, it is not vitiligo‐specific. In contrast, the VitiQoL is disease‐specific and, therefore, more appropriate for vitiligo patients. Unfortunately, it is not freely availabe in all languages. The use of short generic questionnaires, such as *Generalized Anxiety Disorder‐2* (GAD‐2) and *Patient Health Questionnaire‐2* (PHQ‐2), allows for a simple screening for the possible presence of anxiety disorders and depression in routine dermatological care, and should be implemented as part of holistic therapy management of vitiligo as a systemic inflammatory disease.^165^ Contact with self‐help groups or self‐help societies (in Germany, *Deutscher Vitiligo‐Bund* and *Deutscher Vitiligo‐Verein*) may make dealing with the disease easier by enabling the sharing of experiences with other affected people.

The DLQI is routinely used in dermatology and allows for a first screening for psychosocial impairment in vitiligo patients. In contrast to the DLQI, the VitiQoL is disease‐specific and, therefore, more appropriate for vitiligo patients.

### Camouflage

Camouflage is an essential component of the supportive measures in vitiligo. Numerous dermatocosmetic products are available that are characterized by high pigment density and good adhesion and achieve excellent coverage of vitiligo lesions.^166^, ^167^ Self‐tanning products contain dihydroxyacetone (DHA), which reacts chemically with proteins in the *stratum corneum*. As a result, the skin obtains a golden‐brown color that persists for 3–7 days. Although the achieved color can be modified by variation of the DHA concentration, there is often a color mismatch between healthy and DHA‐treated skin.^168^ Permanent make‐up is another option, especially for recalcitrant regions like acral areas, nipples, or lips.^169^ Complications, such as infections, allergic reactions, or scar formation, are rare.^170^ However, the pigmentation effect produced by micropigments is static, and thus satisfactory results cannot always be guaranteed in the course of vitiligo.^171^

Numerous dermatocosmetic products are available that are characterized by high pigment density and good adhesion and achieve excellent coverage of vitiligo lesions.

### Depigmentation

Depigmentation appears to be the last option and is almost exclusively considered in patients with dark skin phototype, subtotal vitiligo and high psychological strain.^172^ Due to the development of new therapies, the goal of irreversibly destroying remaining epidermal melanocytes is becoming less of a focus. Depigmentation of focal residual pigmented skin can be achieved by cryotherapy. Of note, there are no officially approved medicinal products for permanent depigmentation in the German‐speaking region. For this purpose, monobenzylether of hydroquinone (monobenzone), which triggers a chemically induced leukoderma, is usually considered.^173^ The treatment takes place over months and results in marked, but often incomplete depigmentation of the treated skin areas. Repigmentation in previously successfully depigmented skin may also occur.^174^ Concentration‐dependent skin irritation occurs in approximately 50% of treated patients.^173^ Another method for destruction of melanocytes is the Q‐switched ruby laser that can also be used in combination with chemical depigmentation.^175^, ^176^

Depigmentation is the last option and almost exclusively considered in patients with dark skin phototype, subtotal vitiligo and high psychological strain.

### Emerging therapeutic perspectives

Topical and systemic JAK inhibitors are currently the most advanced new substance class in clinical research on patients with vitiligo. Its efficacy as a monotherapy or combination therapy with NB‐UVB is evaluated in many clinical trials worldwide in patients with active and stable NSV. t

Topical and systemic JAK inhibitors are currently the most advanced new substance class in clinical research on patients with vitiligo.

Additional JAK inhibitors currently under clinical evaluation include tofacitinib, ritlecitinib, upadacitinib, baricitinib, povorcitinib, and cerdulatinib (https://classic.clinicaltrials.gov/). Ritlecitinib, a JAK3/TEC tyrosine kinase inhibitor, is already approved in Germany for extensive forms of alopecia areata since late 2023. In an elaborate placebo‐controlled phase IIb trial including 364 patients with exclusively active NSV and involvement of up to 50% BSA, ritlecitinib at a dose of 30 mg or 50 mg per day resulted in significant improvements of F‐VASI after 24 weeks.^177^ A four‐week high‐dose loading period with up to 200 mg ritlecitinib had no additional effect on the primary endpoint (percent change of F‐VASI from baseline). In the 24‐week extension period of this RCT, ritlecitinib (200 mg/50 mg) resulted in accelerated repigmentation of the face. In a subgroup analysis of 65 patients in this study, the cohort treated with ritlecitinib showed a significant reduction in the density of the CD3^+^/CD8^+^ infiltrate and a dose‐dependent suppression of biomarkers of T‐cell activation associated with an upregulation of melanocyte markers in the skin.^178^ In a currently ongoing multicenter phase III trial, ritlecitinib is being tested in patients with extensive NSV, irrespective of disease activity (https://classic.clinicaltrials.gov/). The measurement of clinically relevant endpoints (such as F‐VASI75) and informative PROMs will provide important additional information.

Upadacitinib, which is approved in Germany for the treatment of atopic dermatitis since 2021, also showed dose‐dependent effects in a randomized double‐blind placebo‐controlled phase II dose‐ranging study on 185 patients with extensive NSV (mean T‐VASI: 21.53 and mean F‐VASI: 1.09).^179^ The maximum percentual reduction of F‐VASI was –21.26 (upadacitinib 11 mg per day) and that of T‐VASI –14.27 (upadacitinib 22 mg per day) after 24 weeks. Even after 52 weeks, continued treatment with upadacitinib showed no plateau in the achieved repigmentation. Currently, a multicenter phase III study is being conducted on this JAK1 inhibitor.

Based on case reports and smaller case series, UV exposure seems to enhance the effect of topically or systemically given JAK inhibitors.^180^, ^181^ In a small prospective controlled open‐label study with 33 patients treated with 2 mg baricitinib per day for 16 weeks, the combination with NB‐UVB showed a more pronounced reduction of F‐VASI and T‐VASI compared to NB‐UVB monotherapy. Administration of baricitinib alone was, however, not evaluated.^182^

Another approach for a future therapy of vitiligo is the administration of peptides with melanotropic effect, either alone or in combination with other therapies.

Another approach for a future therapy of vitiligo is the administration of peptides with melanotropic effect, either alone or in combination with other therapies. Afamelanotide (Nle5‐d‐Phe7‐α‐MSH) is a superpotent synthetic analog of α‐melanocyte‐stimulating hormone (α‐MSH) implanted subcutaneously in a *slow‐release* formulation. It is approved for the treatment of erythropoietic protoporphyria since 2015.^183^ Apart from their clinically unequivocal pigmentation effect, α‐MSH and agonists that activate the melanocortin‐1 receptor (MC1R) also have cytoprotective, immunomodulatory, and indirect antioxidative effects^184^, ^185^, ^186^ that are of interest for the treatment of vitiligo. In two small studies, additive monthly administration of afamelanotide was superior to NB‐UVB phototherapy alone, achieving a faster response and higher repigmentation rates. However, the effect was restricted to dark skin phototypes (IV–VI).^187^, ^188^ Currently, the efficacy of afamelanotide as monotherapy in vitiligo patients with skin phototype IV–VI is being tested in clinical trials (https://classic.clinicaltrials.gov/). Recently, promising effects of dersimelagon, the first available, orally administered, highly selective MC1R agonist, were reported in patients with erythropoietic protoporphyria.^189^ Dersimelagon is currently in the clinical approval phase for this *orphan disease*, and as a non‐peptide MC1R agonist, could also be used in future clinical studies on patients with vitiligo. Such MC1R agonists might be particularly suitable for vitiligo patients with darker skin phototypes.

## CONFLICT OF INTEREST STATEMENT

M.B. has received honoraria as advisor and/or lecturer from pharmaceutical companies (AbbVie, Incyte, MSD, Pfizer) developing and selling drugs for treatment of vitiligo. He receives grants from Incyte for conducting a non‐interventional study in patients with vitiligo (VitiligoHealth). A.T. has received honoraria as advisor and/or lecturer from Incyte Biosciences Austria and Incyte International Sàrl.

## CME‐Questions – Lernerfolgskontrolle

1. Welche Aussage zur Ätiopathogenese der Vitiligo trifft zu?
  - Bei der NSV wurde eine Persistenz von Herpesviren in läsionaler Haut gefunden.
  - In der Haut von Patienten mit NSV sind die Spiegel reaktiver Sauerstoffspezies erhöht.
  - Bei der NSV sezernieren Keratinozyten Chemokine, die Th17‐Zellen mit einer IL‐17‐Signatur anlocken.
  - Es besteht eine gestörte Wundheilung bei der NSV.
  - Gedächtniszellen der Haut (T_RM_) werden bei der NSV durch IL‐4 aktiviert.
2. Welche Aussage zur NSV ist richtig?
  - Die Vitiligoherde sind asymmetrisch verteilt.
  - Schleimhäute sind niemals betroffen.
  - Die gemischte Vitiligo wird auch zur NSV gerechnet.
  - Das Gesicht ist niemals isoliert betroffen.
  - Der Verlauf hängt vom Hautphototyp ab.
3. Welche Aussage ist richtig?
  - Die SV ist häufiger als die NSV.
  - Die SV zeigt häufig Rezidive.
  - Die NSV kann häufig schon bei der Geburt vorliegen.
  - Die SV zeigt frühzeitig eine Leukotrichie.
  - Die NSV ist üblicherweise selbstlimitierend.
4. Welche Aussage ist **nicht** richtig? Zu den klinischen Zeichen einer aktiven Vitiligo zählen …
  - Inflammierte Vitiligo
  - Konfetti‐Läsionen
  - Positives Köbner‐Zeichen
  - Trichrom‐Vitiligo
  - Halo‐Naevi
5. Welche Aussage zur topischen Therapie der Vitiligo ist richtig?
  - Topische Calcineurinantagonisten sind topischen Kortikosteroiden überlegen.
  - Statt topischen Kortikosteroiden der Klasse IV sollten eher moderne Klasse‐III‐Präparate benutzt werden.
  - Topische Calcineurinantagonisten können zu Hautatrophie und Teleangiektasien im Gesicht verursachen.
  - Topische Kortikosteroide sollten nicht mit einer Phototherapie kombiniert werden.
  - Viele Studien belegen einen Zusatzeffekt von Antioxidanzien bei topischen Calcineurinantagonisten und Kortikosteroiden.
6. Welche Aussage zur aktuellen Therapie mit Ruxolitinib‐Creme ist korrekt?
  - Topisches Ruxolitinib ist für die SV zugelassen.
  - Kinder mit NSV ab dem 12. Lebensjahr dürfen behandelt werden.
  - Mit Ruxolitinib‐Creme darf eine befallene Körperoberfläche von bis zu 20% behandelt werden.
  - Pruriginöse Hautveränderungen können als Nebenwirkung auftreten.
  - Die Therapiedauer ist auf 12 Monate begrenzt.
7. Welche Aussage zur Phototherapie der Vitiligo trifft zu?
  - Eine generalisierte aktive NSV sollte am besten mit einer gezielten Lichttherapie behandelt werden.
  - Eine NB‐UVB ‐Ganzkörper‐Phototherapie sollte nicht länger als 6 Monate durchgeführt werden.
  - Eine Kombination von NB‐UVB mit TCI verbessert signifikant die Repigmentierung an den Akren.
  - Bestimmte Antioxidanzien können in Kombination mit NB‐UVB die Repigmentierung verstärken.
  - Bei der NB‐UVB‐Ganzkörper‐Phototherapie sollte fünfmal pro Woche bestrahlt werden.
8. Welche Aussage zur Systemtherapie der Vitiligo trifft zu?
  - TNF‐Antagonisten können die Krankheitsaktivität stoppen.
  - Eine OMP‐Therapie der aktiven Vitiligo mit Dexamethason (2 x pro Woche mit 2,5–5 mg) wird üblicherweise über 3–6 Monate durchgeführt.
  - Systemische Kortikosteroide sollten nicht mit einer Lichttherapie kombiniert werden.
  - Systemische Kortikosteroide als Monotherapie führen bei aktiver NSV zu einer deutlichen Repigmentierung.
  - Ritlecitinib ist zur Therapie der aktiven oder rapid progressiven NSV seit November 2023 zugelassen.
9. Welche Aussage ist **nicht** richtig?
  - Der VitiQoL gilt als vitiligospezifisches PROM.
  - Der VES ist ein intuitiver Score zu Erfassung der Ausdehnung der Vitiligo in Prozent BSA und basiert auf Referenzbildern.
  - Der VES kann auch zuverlässig von Patienten mit Vitiligo angewandt werden (SA‐VES).
  - Der VASI errechnet sich aus Ausdehnung und dem jeweiligen Grad der Depigmentierung im Gesicht (F‐VASI) und am ganzen Körper (T‐VASI).
  - Der VETF‐Score ist zur Erfassung der Lebensqualität von Patienten mit Vitiligo der der geeignetste Score.
10. Welche Aussage zu den supportiven Maßnahmen bei Vitiligo ist richtig?
  - *Shared decision making* involviert sowohl Betroffene als auch Behandler und erlaubt eine gemeinsame Therapieentscheidung zugunsten einer verbesserten Compliance und Adhärenz.
  - Zur permanenten Depigmentierung wird eine 10% Hydrochinon‐Creme eingesetzt.
  - Entstigmatisierungsprogramme haben keinen Einfluß auf die psychosoziale Gesundheit erkrankter Personen.
  - Eine Therapie betroffener Patienten kann auch von Selbsthilfegruppen angeboten werden.
  - Grundsätzlich sollten alle Patienten mit Vitiligo eine Vitamin‐D‐Substitution erhalten, da deren Synthese in der Haut gestört ist.

Liebe Leserinnen und Leser, der Einsendeschluss an die DDA für diese Ausgabe ist der 31. October 2025.

Die richtige Lösung zum Thema Dermatologic diseases of the Breast and Nipple in Heft 05/2025 ist: 1c, 2c, 3e, 4a, 5d, 6a, 7e, 8a, 9d, 10c

Bitte verwenden Sie für Ihre Einsendung das aktuelle Formblatt auf der folgenden Seite oder aber geben Sie Ihre Lösung online unter http://jddg.akademie-dda.de ein.
